# Disease of the Antrum of Highmore

**Published:** 1891-11

**Authors:** Leopold Neumann


### Disease of the Antrum of Highmore.

BY LEOPOLD NEUMANN, D.D.S.

Read before the California State Dental Association.

*Mr. President and Gentlemen:—*

The Antrum of Highmore received its name from the discoverer, Nathaniel Highmore (1651). This cavity is situated in the superior maxilla. Its upper wall is formed by the floor of the orbit; the lower by the alveoli of the molar teeth, the roots of which occasionally penetrate it. The outer wall of the nasal cavity is its interior, while its anterior wall is composed of the malar process. Its shape and size vary very much in different individuals, and even the right and left in the same person sometimes do not correspond.

This term, disease of the antrum, (Bosworth) is used to designate the not infrequent complication or concomitant of catarrhal inflammation of the nasal mucous membrane, which is characterized by an inflammatory process in the mucous membrane lining the Antrum of Highmore, which subsequently degenerates into the purulent process, as is the almost invariable rule where a chronic inflammation of the mucous membrane occurs in a closed cavity.

This pus secretion accumulating in the cavity of the antrum makes its exit through the osteum maxillare and escapes into the nasal cavity, giving rise to a more or less profuse discharge from the nose.

The most frequent affections of the lining membrane of the antrum are preceded by inflammation of the nasal passage, or of the periosteum of the molars, bicuspids or cuspids. Bosworth asserts that the catarrhal inflammation of the nose is the result of local conditions which does not in any degree, probably, operate in the antrum; in other words, so-called nasal catarrh is a perversion of the function of the normal respiratory apparatus of the nose, and its causes operate only on those tissues, and have no effect on the delicate membrane lining the antrum.

Zuckerkandl, Schiffers and others take the ground that the most frequent causes of this disease are transmissions from the inflamed nasal passage to the lining membrane of the antrum; while Frankel, Garretson and others claim that this membrane becomes affected through carious teeth, or through inflammation of the periosteum of the roots of the teeth. This difference of opinion is very easily accounted for if we take into consideration the fact that cases arising from defective teeth are treated by the dentists, and those resulting from disorders of the nose by the specialists in that line. But there is no doubt that both decayed teeth and inflammation of the nose are the direct causes of this disease, in which, at the beginning, when the cavity becomes filled with serum or sero-mucous, the pain on the affected side is a dull, heavy one, extending from the alveolar border to the lower part of the orbit, and to the roof of the mouth. The symptoms and appearance of the diseased part are similar to those in cases of deep-seated periostitis, pulsation, nause, and sometimes fever.

Pus will flow, when lying on one side, passing into the throat in such quantities as to induce nausea. The pain increases until the pus makes its escape through the normal opening of the nose, or an artificial one through either the cheek or the alveolar process, when the pain subsides.

After this the discharge of pus continues from this opening sometimes for a short time, and again for years, till the trouble becomes chronic, the disease in the antrum in the meanwhile undergoing no apparent change.

The odor of this pus is often very offensive, and in color is greenish, yellowish, or a dirty white. After the diagnosis has been established, which is greatly facilitated by translumination, and it is found that the trouble is produced by, or arises from, nasal polypi, hypertrophia rhinitis, or nasal inflammation, a straight opening from the alveolar wall, not less than one-eighth of an inch in diameter, should be made at once, and if necessary even a sound tooth should be sacrificed and extracted; for I have found that drilling through the side of the antrum below the inferior turbinated bone is less satisfactory, as it gives the patient the feeling of undergoing a serious operation, and at the same time does not offer the same ready facilities for cleansing. If the cause is a carious tooth it should be extracted and after this the antrum should be thoroughly cleansed by injecting a solution of listerine two or three times daily till a cure is effected, or any of the following can be used :

Sulpho-carbolate of zinc, five grains to the ounce; resorcin, five grains to the ounce; hydro-naphthol, one-half grain to the ounce; nitrate of silver, five grains to the ounce, or a solution of carbolic acid, three grains to the ounce; boracic acid, twenty grains to the ounce, etc.

If carious bone is present it should be removed. To prevent the closure of the artificial opening a silver drainage tube should be inserted and attached to a tooth with a clasp. The tube is to be kept closed with a small cork and when the discharge ceases the orifice should be allowed to close gradually.

*Case No.* 1.—Mr. R. A.; German; aged 42 years; consulted Dr. H. L. Wagner on account of a discharge from the left nostril, and pain on the left side. Pus was discovered coming from the aperture of the antrum. By the use of translumination the left side was found to remain completely dark, while the right showed the reflection of light. An examination of the teeth revealed the fact that the second left molar, which contained a large amalgam filling, proved to have a dead pulp. I placed the patient under the influence of ether, extracted the tooth and drilled into the antrum. On removing the drill a great deal of foul-smelling pus escaped. A silver drainage tube one-eighth of an inch in diameter was inserted and fastened to the first molar by a clasp.

Daily injections of listerine were used and the discharge decreased, the patient leaving for Germany after some three weeks, whence he informed me that it had discontinued entirely and the pain had left him.

*Case No.* 2.—Mrs. R.; aged 25 years. This lady had been confined to her bed for five days with intense pain, which in her opinion was the result of the operation of transplanting a left superior first molar. When on the morning following she found the pain had increased, and her face had begun to swell she called in her dentist who applied some medicine and kept her in bed two days more.

By that time the agony had become insufferable and she insisted that he should extract the tooth, which he did. But the removal of the tooth not having given the hoped-for relief, I was consulted, and found her, as stated before, with a very much swollen face, a pulsating pain extending from the roof of the mouth to the orbit. By probing, a small opening into the antrum was found, through which I passed a small lancet. On removing this it was followed by a discharge of purulent, yellowish, thick pus, which gave immediate relief. On the following day, by using cocaine in the operation, the opening was enlarged with a drill specially made for antrum cases, one-eighth inch in diameter. I then followed the usual course of treatment, effecting a cure in five weeks. At the end of six months the opening had become very small, and after one year had closed completely.

*Case No.* 3.—Mrs. H.; aged 33 years; came to me with the following history of her case: Her dentist had been treating the left canine, which had been filled with gold, and was very sensitive to the touch. There was no swelling, and he followed the usual course outlined in such cases, of taking out the filling and nerve, which allowed a copious escape of odorous pus. After two months of treatment without any apparent improvement he, with a syringe, injected some medicine in the canal. To his astonishment the injection seemed to have disappeared, and after frequent repetitions the solution finally made its way through the left nostril.

When this had continued about a year without improvement the patient insisted on having the tooth extracted and the root, on examination, proved to be honey-comed.

He then drilled from the canine fossa into the antrum and after a year's time, no relief being experienced, the patient came to me.

The discharge was of such a nature that the presence of carious bone was indicated, the removal of which I advised, as also the second bicuspid and the first molar, both of which contained large fillings, and were so-called dead teeth.

After two weeks' treatment, consisting of daily washing out with listerine, though the odor disappeared entirely the discharge has not dimished, and to-day is as large in quantity as when originally seen by me. To prevent the antrum filling with pus, which causes intense pain in the head, it has to be washed out daily, and though in this case every kind of injection known to be efficacious in this disease has been used, success has not yet crowned my efforts.

By the use of the antroscope the mucous membrane was found to be enlarged to about eight times its normal thickness.

The antroscope is an instrument constructed on the principle of the cystoscope and we are indebted to Dr. H. L. Wagner, of this city, for this valuable aid to the examination of the antrum. It can be used only after the antrum has been opened, but then with an electric light the whole cavity can be illumined and its mucous membrane examined. I have operated on twelve cases where the causes were other than from the teeth, and have found among these but one on the right side. Of these twelve cases eight were examined by translumination and the left side was found dark, except in one case in which this system showed light through both sides, and still when the antrum was drilled into it was found to be filled with pus.

*Dr. Van Orden.*—Mr. President: You asked me to open the discussion on this paper. I have not had an opportunity to prepare myself. I did not expect the paper to be read this afternoon. Speaking of the test of illumination it may be well to call attention to a case where illumination was used with the lateral incisor, which is pulpless. I should say that while that method would be, as a rule a test, there might be cases which would not answer to that test. The degree of density of pus, I should state, would sometimes be so near that of the tissues, that transIumination would not show any line of demarcation. In the case of the pulseless lateral incisor, in which I used the electric method, I examined the case a number of times and utterly failed to detect any change of color. The case continued to trouble, so that at last I was obliged to test the vitality of the tooth by drilling into the pulp chamber, and found that the canal was empty; so that it brings in the practical points in regard to the treatment of pulpless teeth. There was one remedy not mentioned—that is the chloride of zinc. It would be, my judgment theoretically, that cases would yield to treatment by chloride of zinc injections as readily as to any medicament. As to the dimentions of the antrum, while in the main the roots of the molar teeth are concerned and have some relationship to it, it may extend to the laterals. Perhaps it seldom extends to the centrals. I remember my great surprise when a very young practitioner in treating an open abscess through the lateral incisor and penetrating the antrum. It yielded very readily in a few days by the treatment of the lateral and a few injections into the antrum. It is to be noted that the dimensions of the antrum increase with age. It is much smaller in children than in adults, and the wall becomes very thin sometimes in old age—very thin indeed, to the point that it is very readily perforated. As to the perforation of the antrum, it is often preferable to resort to amputation of one of the roots of a superior molar instead of the removal of the tooth. As Dr. Neumann has stated the opening should be an eighth of an inch, at least, in diameter. The removal of the root would obviate that and a valuable tooth might often be saved. It is a question among specialists whether treatment through the root is always a necessary thing. One authority goes so far in his statement of cases as to close up openings that have been made by dentists, going on with the treatment of the catarrhal condition of the nose, and in a majority of cases with success. He cites a very large number of cases in which the alveolar opening was kept and which would not yield to treatment.

